# Taming Superbugs: Current Progress and Challenges in Combating ESKAPE Pathogens

**DOI:** 10.3390/pathogens15010028

**Published:** 2025-12-24

**Authors:** Helal F. Hetta, Fatma R. Khalaf, Ahmed A. Kotb, Marah N. Alatawi, Abdullah S. Albalawi, Ahmad A. Alharbi, Maryam K. Aljohani, Shumukh Saad Aljohani, Majd S. Alatawi, Noura H. Abd Ellah, Basem Battah, Matthew G. Donadu, Vittorio Mazzarello

**Affiliations:** 1 Division of Microbiology, Immunology and Biotechnology, Department of Natural Products and Alternative Medicine, Faculty of Pharmacy, University of Tabuk, Tabuk 71491, Saudi Arabia; hhussen@ut.edu.sa (H.F.H.); aam_alharbi@ut.edu.sa (A.A.A.); 2 Primary Health Care Department, Faculty of Nursing, Al-Ahliyya Amman University, Amman 19111, Jordan; f.khalaf@ammanu.edu.jo; 3 Family and Community Health Nursing Department, Faculty of Nursing, Assiut University, Asyut 71515, Egypt; 4 Department of Microbiology and Immunology, Faculty of Pharmacy, Assiut University, Assiut 71515, Egypt; ahmedabedelaziz994@aun.edu.eg; 5 Department of Medical Laboratory Technology, Faculty of Applied Medical Sciences, University of Tabuk, Tabuk 71491, Saudi Arabia; m.nalatawi@ut.edu.sa; 6 Department of Pharmaceutical Chemistry, Faculty of Pharmacy, University of Tabuk, Tabuk 71491, Saudi Arabia; abs_albalawi@ut.edu.sa; 7 PharmD Program, Faculty of Pharmacy, University of Tabuk, Tabuk 71491, Saudi Arabia; 451003177@stu.ut.edu.sa (M.K.A.); 451004253@stu.ut.edu.sa (S.S.A.); 451003956@stu.ut.edu.sa (M.S.A.); 8 Department of Pharmaceutics, Faculty of Pharmacy, Assiut University, Assiut 71515, Egypt; nora.1512@aun.edu.eg; 9 Department of Biochemistry and Microbiology, Faculty of Pharmacy, Antioch Syrian Private University, Maaret Siadnaya 36822, Syria; basem.battah.sc@hotmail.com; 10 Hospital Pharmacy, Giovanni Paolo II Hospital, ASL Gallura, 07026 Olbia, Italy; mdonadu@uniss.it; 11 Department of Medicine, Surgery and Pharmacy, Scuola di Specializzazione in Farmacia Ospedaliera, University of Sassari, 07100 Sassari, Italy; 12 Department of Biomedical Sciences, University of Sassari, 07100 Sassari, Italy

**Keywords:** ESKAPE pathogens, multidrug resistance, vaccine targets, antigen discovery, sustainability, mRNA vaccines, biofilm, OMVs

## Abstract

The global incidence of multidrug-resistant (MDR) ESKAPE pathogens—comprising *Enterococcus faecium*, *Staphylococcus aureus*, *Klebsiella pneumoniae*, *Acinetobacter baumannii*, *Pseudomonas aeruginosa*, and *Enterobacter* species—has surged alarmingly in recent years, posing a significant challenge to healthcare systems worldwide. These organisms are notorious for their capacity to evade the effects of multiple classes of antibiotics, leading to treatment failures, increased morbidity and mortality, and escalating healthcare costs, all of which have placed unprecedented strain on existing infection control measures. This review encapsulates the progress in target-driven vaccine research, including the genomic discovery of highly conserved surface antigens, iron acquisition systems, biofilm- and quorum-sensing-related proteins, and computationally predicted epitopes, which are considered the most attractive targets for broad-spectrum vaccination. Novel vaccine platforms, such as outer membrane vesicles (OMVs), mRNA technologies, and multi-epitope constructs, will rapidly drive the translation of these targets into next-generation vaccine formulations. Nevertheless, challenges such as antigenic variation and immune evasion, as well as the need for a robust mucosal and cross-protective immune response, persist. The sustainability in interdisciplinary investigations are required, along with adjunctive measures and investment in the development of advanced discovery and delivery systems, to achieve the ultimate goal of successful vaccines against MDR ESKAPE infections and to mitigate the worldwide burden of antimicrobial resistance.

## 1. Introduction

Antimicrobial resistance (AMR) has emerged as one of the most urgent global health threats of the 21st century. Recent WHO estimates indicate that more than 1 million deaths each year are directly attributable to bacterial AMR, with an additional ~5 million deaths associated with resistant infections worldwide [1]. Confirming that the 2024 GRAM–Lancet analysis that reported about 4.71 million deaths in 2021 were associated with bacterial AMR, of which 1.14 million deaths were directly attributable to resistant infections [2].

The ESKAPE pathogens—*Enterococcus faecium* (*E. faecium*), *Staphylococcus aureus* (*S. aureus*), *Klebsiella pneumoniae* (*K. pneumoniae*), *Acinetobacter baumannii* (*A. baumannii*), *Pseudomonas aeruginosa* (*P. aeruginosa*), and *Enterobacter* spp.—constitute a formidable threat to global health as they are primary contributors to multidrug-resistant (MDR) and healthcare-associated infections (HAIs) worldwide. In 2019 alone, these pathogens were related to ~1.27 million fatalities worldwide [3,4].

Multidrug resistant (MDR) Gram-negative ESKAPE are within the “Priority 1: Critical” pathogen group on the WHO’s priority pathogens list, while MDR Gram-positive ESKAPE; *E. faecium* and *S. aureus* are classified as “Priority 2: High”, pathogens according to WHO priority list of antibiotic-resistant bacteria due to their high virulence, evolving resistance profiles, and significant healthcare burden [5,6].

The clinical burden of ESKAPE pathogens is not limited only to morbidity and mortality; it also has significant economic implications in terms of extended hospitalization and higher costs of healthcare. The continual increase in resistance and the lag in bringing new antibiotics to market reinforce the urgency for alternative approaches, such as effective vaccines, to reduce the incidence of infection and the dependence on antimicrobial therapies [7,8,9,10,11,12].

Vaccine discovery against ESKAPE organisms is urgently needed, yet it faces multiple challenges. The antigenic diversity, both intra- and inter-species, the numerous and redundant virulence factors, immune evasion strategies, and the complex pathogenesis of these pathogens have precluded the successful development of conventional vaccines. Moreover, the variety of clinical forms and strain heterogeneity necessitate the development of vaccines that can induce cross-protection against different species and strains [13,14].

To address this problem, the recent trend in vaccine design has shifted toward a target-based approach. This strategy is centered on the conserved molecular targets shared by the ESKAPE organisms, rather than focusing on the individual organisms themselves. Priority antigens include surface-exposed adhesins and colonization factors, iron-acquisition systems important for bacterial survival in the host, secreted toxins, biofilm-associated antigens that promote evasion and tolerance of antibiotics, and new technologies for antigen delivery, such as mRNA vaccines and OMVs [13,14,15,16,17,18].

Novel vaccine platforms, specifically the mRNA platform, have emerged as favored vaccine candidates due to their rapid development, potential to elicit strong cellular and humoral immunity, and versatility in accommodating antigen variability. By the same token, OMVs provide a potential delivery vehicle capable of displaying several antigens in their native conformations, thereby ensuring their immunogenicity and allowing for the development of multivalent vaccine formulations. Machine learning and bioinformatics methods are also being used to expedite antigen identification and to predict candidate vaccines that are highly protective against ESKAPE pathogens [15,19,20,21]. This review focuses on current literature and future directions in developing vaccines that target conserved antigens that overcome the rapidly growing threat posed by ESKAPE pathogens.

## 2. Challenges Against Bacterial Vaccine Development

### 2.1. Antigenic Diversity and Immune Evasion

High genetic and immunological diversity of nosocomial bacterial pathogens, including variable surface proteins and polysaccharides, complicates vaccine design and limits cross-protection [22]. Their biological complexity and genetic diversity hinder the development of vaccines targeting multidrug-resistant ESKAPE pathogens. A major barrier is the high genetic plasticity of ESKAPE pathogens, driven by mutation and horizontal gene transfer via mobile genetic elements, resulting in remarkable antigenic diversity, exemplified by over 80 capsular serotypes in *K. pneumoniae* and multiple strain-specific adhesins and immune evasion proteins in *S. aureus* [15]. Functional redundancy of virulence factors, such as multiple adhesins in *S. aureus* (e.g., ClfA, ClfB, FnBPs), necessitates multivalent vaccines targeting several antigens simultaneously [15].

Immune evasion strategies, including antigenic variation, biofilm formation, and the production of immunomodulatory toxins, undermine vaccine efficacy. For instance, *P. aeruginosa* biofilms inhibit opsonophagocytosis, while *A. baumannii* secretes vesicles that modulate host immunity [15]. To overcome antigenic diversity, vaccine efforts increasingly focus on conserved antigens identified through integrated genomic, proteomic, and immunoinformatic approaches, such as the zinc-binding lipoprotein AdcA in *S. aureus* and *E. faecium*, and outer membrane proteins like OmpK36 in *K. pneumoniae* [15,20].

### 2.2. Pathogen-Specific and Cross-Species Obstacles

Extensive strain-to-strain variability, even within a single species, complicates the development of cross-protective vaccines. Antigenic variability among clinical isolates limits the breadth of protection that a single-antigen vaccine can provide. In Gram-positive bacteria, such as *S. aureus* and *E. faecium*, strain-specific surface structures and immune evasion proteins complicate the development of universal vaccine strategies [22]. For Gram-negative organisms, OMV-based platforms provide multivalent antigen presentation in their native conformation; however, achieving cross-strain protection remains challenging due to variations in outer membrane constituents and lipopolysaccharide (LPS) structures [23]. Antigen selection must thus carefully balance strain specificity and conservation, with recent efforts leveraging reverse vaccinology and multi-epitope design to overcome this obstacle [22].

### 2.3. Clinical and Strain Heterogeneity

Broad heterogeneity in clinical manifestations, infection routes, and host responses further complicates vaccine efficacy and trial design. The antigenic composition of ESKAPE pathogens can vary widely among clinical isolates, which impacts the performance of both subunit and OMV-based vaccines. This variability is particularly problematic for platform technologies, such as mRNA vaccines, which can rapidly adapt. Still, antigen selection must be based on a detailed understanding of conserved targets among diverse clinical strains. Moreover, delivery system optimization remains a challenge across all ESKAPE species, due to differences in immune niche targeting (e.g., respiratory vs. urinary tract pathogens) [22]. Broad heterogeneity in clinical manifestations, infection routes, and host responses further complicates vaccine efficacy and trial design. The antigenic composition of ESKAPE pathogens can vary widely among clinical isolates, which impacts the performance of both subunit and OMV-based vaccines. This variability is particularly problematic for platform technologies, such as mRNA vaccines, which can rapidly adapt. Still, antigen selection must be based on a detailed understanding of conserved targets among diverse clinical strains. Moreover, delivery system optimization remains a challenge across all ESKAPE species, due to differences in immune niche targeting (e.g., respiratory vs. urinary tract pathogens) [22].

## 3. Emerging Vaccine Platforms and Technologies

### 3.1. mRNA-Based Vaccines

**Figure 1:**
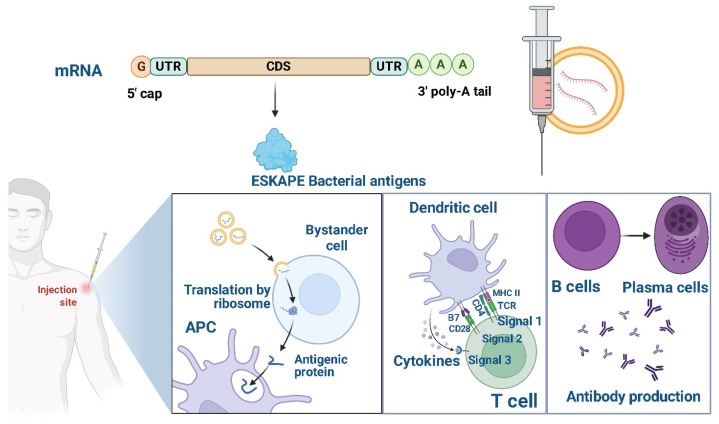
mRNA Vaccine Mechanism for Inducing Immunity Against ESKAPE Pathogens. This diagram illustrates the mechanism of mRNA vaccines encoding ESKAPE bacterial antigens. Upon intramuscular injection, lipid nanoparticle-encapsulated mRNA is delivered into host cells, where it is translated into antigenic proteins by host ribosomes. Antigen-presenting cells (APCs), such as dendritic cells, uptake and process these antigens, presenting them via MHC class II molecules to CD4+ T cells. This interaction, alongside co-stimulatory signals (e.g., B7-CD28 binding) and cytokine signaling, activates T cells. In parallel, B cells are stimulated and differentiate into plasma cells, resulting in the production of antigen-specific antibodies. This approach leverages the adaptability and rapid design potential of mRNA technology to elicit protective immune responses against diverse and multidrug-resistant ESKAPE bacterial pathogens. (Created with BioRender.com).

mRNA vaccines represent a cutting-edge technology with remarkable flexibility and adaptability for targeting MDR ESKAPE pathogens, which are major causes of hospital-acquired infections. The core of this technology is the rational design of the mRNA molecule [24], as illustrated in Figure 1, which comprises a 5′ cap, untranslated regions (UTRs), a coding sequence (CDS), and a poly(A) tail. Each element can be optimized to enhance mRNA stability, translation efficiency, and immunogenicity. For example, modifications to the 5′ cap, such as the Cap1 and Cap2 structures, and fine-tuning of UTRs prolong antigen expression, which is crucial for effective immune responses against persistent bacteria [25,26,27,28,29]. Codon optimization within the CDS ensures robust antigen production in host cells, while the poly(A) tail stabilizes the mRNA, extending its half-life and supporting sustained protein synthesis [19,30,31,32].

The versatility of mRNA vaccines enables the encoding of diverse bacterial antigens, including outer membrane proteins (OMPs), virulence factors, and antibiotic resistance genes. For instance, vaccines targeting *K. pneumoniae* can express OMPs or resistance determinants to prime immune recognition. Similarly, vaccines against *P. aeruginosa* focus on the OprF and OprI proteins. At the same time, those for *A. baumannii* target OmpA or Omp22, all of which are critical for bacterial adhesion, immune evasion, and biofilm formation. Incorporating peptide linkers like Gly-Gly-Gly-Ser enables inclusion of multiple antigenic targets in a single construct, enhancing immunogenicity and protection breadth [19,29,30].

Efficient delivery of mRNA into host cells is achieved using advanced systems such as lipid nanoparticles (LNPs) and polymeric nanoparticles (NPs). LNPs encapsulate mRNA, protecting it from degradation and facilitating cellular uptake and endosomal escape. Polymeric NPs, such as PLGA and chitosan-based particles, have been engineered for targeted delivery to specific tissues or immune cells and have shown promising results in preclinical models against pathogens like *S. aureus* and *P. aeruginosa*. The route of administration-intramuscular, intradermal, subcutaneous, or intranasal-can be tailored to optimize immune responses, including mucosal immunity for respiratory infections caused by *A. baumannii* [19,32].

Adjuvants further enhance immunogenicity, with natural options such as saponins and chitosan, as well as chemical adjuvants like alum and CpG motifs. For example, saponins have been shown to boost immune responses against *K. pneumoniae*, while CpG motifs have enhanced T-cell activation in vaccines targeting *E. faecium*. These advances enable rapid, precise development of mRNA vaccines tailored to the challenges posed by ESKAPE pathogens [19,30].

The mRNA vaccine platform offers several advantages in combating MDR ESKAPE pathogens. Its modularity allows for rapid design and production, making it adaptable to emerging threats. Encoding multiple antigens into a single construct broadens protection and reduces the risk of immune escape. mRNA vaccines are non-infectious, do not integrate into the host genome, and their cell-free production avoids handling live pathogens, streamlining manufacturing and reducing biosafety risks. Advanced delivery systems and potent adjuvants elicit strong humoral and cellular immunity essential for clearing resilient bacterial infections [19,30,31,32].

However, challenges remain. The rapid evolution of escape pathogens demands continual antigen discovery and vaccine updates. Optimizing mRNA structure and delivery vehicles requires balancing stability, translation efficiency, and immunogenicity to achieve optimal results. Although LNPs and polymeric NPs are effective, further research is necessary to enhance their specificity, minimize toxicity, and ensure consistent performance across diverse populations. Adjuvant selection must maximize efficacy while minimizing side effects. Translating preclinical success into clinical efficacy is a significant challenge, requiring large-scale trials to confirm safety and effectiveness, particularly in high-risk populations. Regulatory pathways for bacterial mRNA vaccines are still evolving, which may potentially delay their approval. Additionally, long-term durability of immune responses and booster requirements remain under investigation [19,30,31,32].

This type of vaccine offers flexibility for encoding *E. faecium* antigens such as the conserved AdcA protein, which has demonstrated cross-protection against Gram-positive ESKAPE pathogens. These RNA vaccines can be rapidly designed to induce both B- and T-cell responses, though clinical translation is still pending [33].

mRNA vaccines targeting conserved *S. aureus* antigens, including AdcA and other surface molecules, show promising reactivity in preclinical studies. These vaccines are designed to elicit both antibody and cytotoxic T-cell responses, potentially overcoming the limitations of previous protein subunit vaccines. A critical challenge in developing such vaccines is achieving broad coverage across different clinically observed manifestations and diverse *S. aureus* strains [34,35,36].

It can also directly encode surface antigens of K. pneumoniae, such as OmpK36 and capsule surface proteins, and can be rapidly tailored to new resistant strains. The main challenge remains selecting antigens that confer significant cross-protection due to the wide serotype diversity of *K. pneumoniae* [37].

Research on this type of vaccine for *A. baumannii* is ongoing, targeting conserved OMV components or surface-exposed proteins. These vaccines aim to induce robust humoral and cellular immune responses, although they remain in early stages of clinical development [38].

mRNA vaccines targeting *P. aeruginosa* antigens, including OprF and PcrV, are under investigation and have shown potential to induce strong cellular and humoral immune responses. However, challenges remain in antigen selection and delivery due to the diversity of clinical isolates, which has hindered vaccine progress [39,40].

The inherent flexibility of mRNA platforms also may enable quick adaptation for the expression of newly discovered *Enterobacter* antigens; however, this is currently in the research phase [41].

### 3.2. OMV-Based Vaccines (Outer Membrane Vesicles)

**Figure 2:**
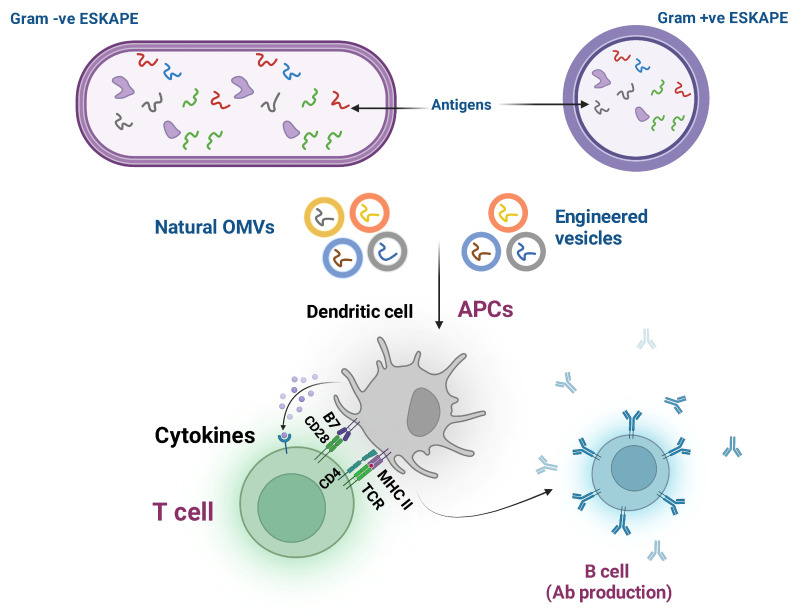
Antigen Delivery and Immune Activation via Outer Membrane Vesicles (OMVs) from ESKAPE Pathogens. This schematic illustrates the use of natural and engineered outer membrane vesicles (OMVs) derived from Gram-negative and Gram-positive ESKAPE pathogens for vaccine development. Bacterial cells contain a diverse array of antigens, including surface proteins and polysaccharides. These antigens are incorporated into natural OMVs or engineered vesicles, which are taken up by antigen-presenting cells (APCs), such as dendritic cells. APCs process and present antigens via MHC molecules to T cells, initiating an adaptive immune response. Cytokine signaling activates B cells, promoting antibody (Ab) production. OMV-based platforms offer multivalent antigen presentation and mimic native bacterial components, making them promising tools for eliciting broad immune responses against multidrug-resistant ESKAPE pathogens. (Created with BioRender.com).

OMV vaccine development against ESKAPE pathogens is an emerging approach that has gained focus recently. For *E. faecium*, this approach is challenging because OMV production is typically associated with Gram-negative bacteria, whereas *E. faecium* is a Gram-positive bacterium. However, proteomics and antigen discovery have identified conserved surface proteins that may be incorporated into OMV-mimicking platforms to enhance immunogenicity, as indicated in Figure 2. Studies show that extracellular membrane vesicles (MVs) from *E. faecium* contain protein candidates that, when used to immunize animals, elicit strong immune responses and opsonophagocytic killing against multiple strains, indicating MVs as promising multi-antigen vaccine platforms for multidrug-resistant *E. faecium* [33]. The natural production of OMVs is scarce in Gram-positive bacteria like *S. aureus*; however, engineered vesicle-like particles carrying *S. aureus* antigens are attractive vaccine platforms because they can deliver multiple surface and secreted proteins in their native configuration, aiding in eliciting an immune response. These approaches aim to mimic the immunogenic advantages of OMVs despite the lack of natural OMV production in Gram-positive strains [34,35].

Although *S. aureus* is a Gram-positive bacterium, the applicability of outer membrane vesicle (OMV)-based vaccination has recently been demonstrated using *Escherichia coli* engineered with plasmids encoding five staphylococcal antigens, each fused to the leader sequence of the endogenous lipoprotein LPP to facilitate antigen trafficking into OMVs. Purified OMVs were successfully employed to immunize mice against *S. aureus*-induced sepsis, kidney abscesses, and skin infections [42]. Interestingly, OMVs derived from *E. coli* lacking staphylococcal antigens also conferred substantial protection in models of sepsis and kidney abscess. This observation may reflect the short interval (10–14 days) between the final immunization and challenge, during which residual immune potentiators elicited by OMV immunization mediate anti-staphylococcal defense. Alternatively, these results may suggest that OMVs themselves can induce non-specific protective immunity, a phenomenon known as innate immune memory or trained immunity, which has previously been demonstrated to confer protection in murine models of *S. aureus* infection [43,44,45,46].

As an alternative to OMVs, which are inherently derived from Gram-negative bacteria, the discovery that Gram-positive bacteria, including *S. aureus*, secrete extracellular vesicles (EVs) has prompted their experimental evaluation as a novel vaccination platform [47,48]. Proteomic analyses have revealed that these EVs are compositionally complex and functionally heterogeneous, encompassing cytoplasmic, secreted, and membrane-associated proteins involved in cellular homeostasis, immune evasion, and antibiotic resistance [47,49]. Analogous to OMVs, *S. aureus* EVs exhibit intrinsic adjuvant properties, as evidenced by their ability to stimulate the production of proinflammatory cytokines such as TNF-α, IL-6, and IL-12 in dendritic cells and dermal fibroblasts [50,51]. Consequently, EVs represent a promising platform for standalone vaccine development. In murine infection models, mice immunized thrice with *S. aureus* EVs demonstrated protective immunity against subsequent pneumonia as early as one week following the final vaccination. Importantly, long-term protective immunity was observed in a lethal *S. aureus*-induced sepsis model 40 days post-final immunization, confirming the induction of durable immune memory [50]. Notably, this immunity was predominantly T cell-mediated and was completely abrogated upon genetic deletion of IFN-γ, consistent with previous studies highlighting T cell-derived IFN-γ as a critical mediator of protection during systemic *S. aureus* infections in both humans and murine models [52,53]. Furthermore, exosomes secreted by mammalian cells have recently been reported to confer protection against *S. aureus* and other bacterial pathogens by acting as decoys for Hla and additional bacterial toxins [54].

The natural outer membrane vesicles (OMVs) of *K. pneumoniae* are loaded with outer membrane proteins and polysaccharides, inherently serving as multiple antigens for the immune system. Recombinant OMVs have demonstrated significant immunogenicity and protective efficacy in animal models, and modifications of lipopolysaccharides (LPS) have optimized their safety profiles [21,55,56,57].

Outer membrane vesicles (OMVs) of *A. baumannii* contain multiple immune proteins, such as OmpA and BauA, and have shown effectiveness in preclinical respiratory infection models when administered intranasally. Engineering strategies, including lipopolysaccharide (LPS) detoxification, have been employed to improve the safety and immunogenicity of these OMVs [38,58,59].

*P. aeruginosa*-derived outer membrane vesicles (OMVs) are inherently immunogenic as they contain multiple antigens such as OprF, OprI, and exotoxins. Animal model studies have demonstrated the efficacy of OMV vaccines, and genetic modifications are employed to improve their safety and antigenicity [39,60,61,62,63]. Vaccine development based on OMVs for *Enterobacter* spp. is emerging, but proteomics profiling and antigen discovery are revealing OMV-associated proteins as potential antigens for future vaccine applications.

### 3.3. Multi-Epitope/Epitope-Based Vaccines

**Figure 3:**
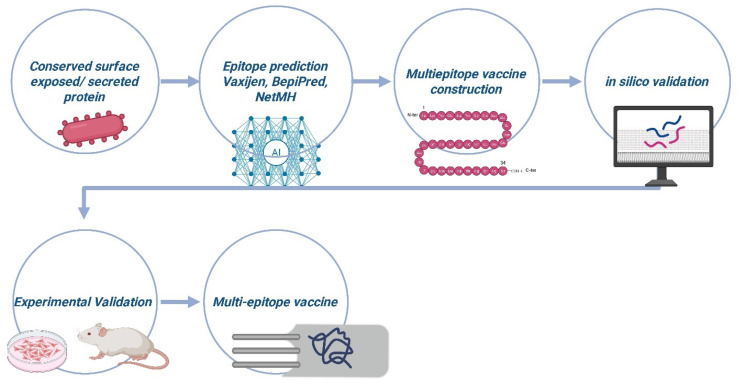
Workflow of Multi-Epitope Vaccine Design Using Immunoinformatics Approaches. This schematic outlines the computational and experimental pipeline for developing multi-epitope vaccines against bacterial pathogens. The process begins with the identification of conserved, surface-exposed or secreted proteins. These candidate antigens are subjected to epitope prediction using tools like VaxiJen, BepiPred, and NetMHC to identify potential B- and T-cell epitopes. Selected epitopes are assembled into a multi-epitope vaccine construct, which is then evaluated through in silico validation to assess immunogenicity, allergenicity, and structural stability. Following computational validation, the construct undergoes experimental validation in vitro and in animal models. Upon successful testing, the final multi-epitope vaccine is prepared for further preclinical development. (Created with BioRender.com).

Multi-epitope-based vaccine development against ESKAPE pathogens leverages bioinformatics and computational tools to accelerate the identification of promising antigens and epitopes, design multi-epitope constructs, and streamline validation. The process, as indicated in Figure 3, begins by selecting conserved, surface-exposed, or secreted bacterial proteins as antigens, utilizing machine learning tools such as VaxiJen and Vaxign2 to evaluate antigenicity, allergenicity, toxicity, and minimize autoimmunity risks. B-cell and T-cell epitopes are then predicted using platforms such as BepiPred, ABCpred, and NetMHCpan, with selection criteria focusing on MHC binding affinity, strain conservation, and broad population coverage. These epitopes are assembled into a vaccine construct, often enhanced with molecular adjuvants and optimized linkers for improved immune response and stability. In silico validation is performed to assess antigenicity, safety, and physicochemical properties, followed by structural modeling and molecular docking to predict interactions with immune receptors. Codon optimization ensures efficient expression in the chosen host. Once validated computationally, the construct is synthesized, expressed, and purified, then tested in vitro and in animal models for immunogenicity, safety, and efficacy against ESKAPE pathogens [14].

Epitope-based vaccines offer significant advantages over traditional platforms, including reduced side effects, targeted immune responses, and streamlined manufacturing. Their modular design enables the formulation of DNA, mRNA, or protein, as well as the inclusion of multiple epitopes, thereby broadening protection and minimizing immune escape. However, challenges remain in accurately predicting epitopes, integrating vaccine components, and conducting limited experimental validation. The inconsistency of computational tools, the lack of standardized assembly methods, and regulatory uncertainties further complicate the development process. Advances in artificial intelligence, integrated design platforms, innovative preclinical models, and clearer regulatory guidelines are crucial to overcoming these barriers. Nevertheless, epitope-based vaccines hold great promise for combating multidrug-resistant ESKAPE pathogens, provided these ongoing challenges are addressed [14].

State-of-the-art vaccine research incorporates computational and immunoinformatics technologies, including in silico epitope selection and prediction, reverse vaccinology, and machine learning, to advance the rational design of multi-epitope vaccines. It can be used to identify and design multiple epitope vaccine of immunodominant epitopes from surface proteins like AdcA and Esp in E. faceium, B and T cell epitopes from conserved *S. aureus* surface proteins, outer membrane and capsule antigens such as Conserved OmpA in Gram-negative ESKAPE, and conserved surface and secreted proteins of *P. aeruginosa* and integration of machine learning enables the design of broad spectrum vaccine less prone to immune escape [33,37,39,64,65,66,67,68]. For epitope-based vaccines, Conserved epitopes have been recognized through machine learning and reverse vaccinology from outer membrane proteins and various other virulence factors, validating the multi-epitope vaccine approach [41,69,70].

Vaccine efforts for *S. aureus* have shifted toward epitope-based strategies, focusing on B- and T-cell epitopes from major antigens, including enterotoxin B, MntC, alpha-toxin, and IsdB. Several B-cell epitopes have demonstrated strong IgG responses and protective immunity in animal models, and some have shown partial protection in clinical and preclinical studies. Multi-epitope constructs (e.g., MAP27, rFSAV) can elicit both humoral and cellular immunity, though not all have translated to robust protection in vivo. Recent advances include the phase 2 clinical trial evaluation of a five-antigen vaccine and monoclonal antibody approaches; however, further optimization and validation are still needed. Some epitope-based vaccines failed to induce protective antibodies, highlighting the challenge of translating immunogenicity into effective immunity [14,71,72,73,74,75,76].

For *K. pneumoniae*, OMPs such as OmpA, OmpK36, and OmpW are attractive vaccine targets due to their conservation and immunogenicity, with OMV-based and recombinant vaccines showing protective efficacy in animal models. Multi-epitope vaccines, such as r-AK36 and mHla-EpiVac, combine epitopes from OMPs and other virulence factors, inducing robust humoral and cellular responses and reducing bacterial burden in preclinical models. Fimbrial adhesin proteins, such as MrkD, have also been targeted; however, their protective efficacy remains to be fully established. Numerous multi-epitope constructs have been predicted using bioinformatics, but most lack in vivo validation. The main challenge remains the antigenic diversity of surface proteins and the need for broad-spectrum protection [14,37,77,78,79,80].

In *P. aeruginosa*, epitope-based vaccines have targeted outer membrane proteins (OMP) such as OprF and OprI, type IV pili components (PilA and PilY1), and type 3 secretion system proteins (PcrV). Several linear B-cell epitopes and multi-epitope constructs have demonstrated protective immunity in animal models, reducing bacterial load and conferring both preventive and therapeutic effects. Nanoparticle delivery systems and fusion constructs have further enhanced immunogenicity. As with *K. pneumoniae*, many bioinformatically predicted multi-epitope vaccines await experimental validation and clinical testing [81,82,83,84,85,86,87,88,89,90].

No *E. faecium* vaccine has reached clinical trials. Preclinical research focuses on conserved surface proteins (e.g., AdcA) and multi-epitope constructs identified through reverse vaccinology and immunoinformatics. These strategies aim to overcome antigenic variability and redundancy, which have historically limited vaccine efficacy [16,65].

## 4. Computational Antigen Discovery and Reverse Vaccinology

Recent advances in computational biology, immunoinformatics, and proteogenomics enable systematic identification of vaccine candidates with enhanced specificity, safety, and cross-protective efficacy. Integrating multi-omics datasets, whole-genome sequencing (WGS), transcriptomics (RNA-seq), ribosome profiling (Ribo-seq), and mass spectrometry-based immunopeptidomics enables the identification of pathogen-derived peptides presented by host immune molecules, such as human leukocyte antigen (HLA). Proteogenomic pipelines further expand antigen discovery by including canonical and non-canonical antigens from previously unannotated genomic loci. Structure- and physics-guided computational models, empowered by artificial intelligence (AI) and machine learning (ML), predict B and T cell epitopes with enhanced immunogenicity and reduced cross-reactivity, enabling rational design of antigens and antibodies to optimize binding affinity, stability, and immune engagement. These computational pipelines also rank conserved antigens across multiple ESKAPE species, facilitating the design of cross-genus protective vaccines [15,19,20,91].

The development of fusion epitope-based vaccines follows a multi-stage computational and experimental pipeline. The process begins with target antigen selection, where conserved and immunogenic antigens are screened from bacterial proteomes using bioinformatics platforms such as VaxiJen, Bowman–Heinson, and Vaxign2, integrating parameters including subcellular localization, transmembrane helices, signal peptides, virulence, allergenicity, and toxicity, with host homology excluded through BLASTp analysis to minimize autoimmunity. Subsequently, epitope prediction and selection is carried out to identify B-cell epitopes (LBLs and CBLs) using BepiPred, ABCpred, IEDB B-cell epitopes, ElliPro, and DiscoTope, and T-cell epitopes (CTLs and HTLs) using IEDB MHC-I binding, IEDB MHC-II binding, NetMHCpan, NetMHCIIpan, and the direct prediction tool CTLPred. Epitope suitability is strengthened by population coverage and conservancy assessment using IEDB Population Coverage, IEDB Epitope Conservancy, IEDB-Clustering, and BLAST [14].

Selected epitopes are then assembled into a multi-epitope vaccine construct incorporating immunostimulatory adjuvants such as CTB, LTB, RplL, Ply, and hBD3, and connected with rationally designed linkers (flexible, rigid, or cleavable, e.g., GGGGS, AAY, GPGPG, EAAAK) generated using SynLinker. The physicochemical properties (antigenicity, allergenicity, toxicity, hydrophilicity, stability, solubility) of the designed construct are assessed through ProtParam, and the tertiary structure is predicted using AlphaFold, SWISS-MODEL, I-TASSER, or Raptor-X. Structural validation includes molecular docking with immune receptors (TLR2 and TLR4) using PatchDock, AutoDock Vina, and HDOCK, followed by molecular dynamics simulations using GROMACS or AMBER to evaluate complex stability parameters such as binding free energy, RMSD, and RMSF. Immune response potential is further evaluated in silico using C-ImmSim and IEDB Class I Immunogenicity. Codon optimization for expression in heterologous hosts is subsequently conducted using JCat or GenSmart before cloning into an expression vector and purification through affinity chromatography (His/GST-tag, Ni-NTA/GST columns) [14].

Finally, in vitro and in vivo verification assesses biosafety (cytotoxicity, hemolysis, tissue lesions) and immunogenicity (IgG/IgA/IgE titers, cytokine secretion, proliferation assays such as MTT/CCK-8, and immune cell differentiation via flow cytometry). Protective efficacy is evaluated in multiple animal challenge models, including pneumonia, sepsis, bacteremia, skin infection, and UTI, in addition to OPK assays and survival analysis. Vaccine constructs demonstrating strong immunogenicity and protection progress to clinical evaluation to determine safety and efficacy in humans [14].

Reverse vaccinology has significantly evolved with the integration of artificial intelligence and machine learning, enabling more accurate antigen discovery and prioritization. Among these advancements, VacSol-ML(ESKAPE) represents a novel AI-driven RV framework specifically designed for the prediction of protective antigens against ESKAPE pathogens. The tool employs a comprehensive ML pipeline incorporating curated BPAg datasets, autoencoder-based feature extraction, and hyperparameter-optimized supervised classifiers, validated through stratified 5-fold cross-validation and independent dataset benchmarking. Notably, VacSol-ML(ESKAPE) is the first platform trained exclusively on ESKAPE-derived protective and non-protective proteins, and it demonstrates superior predictive performance—achieving the highest precision, recall, weighted F1 score, and MCC when compared with existing ML- and rule-based antigen prediction tools. This focused and robust predictive capability highlights its utility as a promising asset in accelerating vaccine design against multidrug-resistant pathogens [20].

For Gram-positive pathogens such as *S. aureus* and *E. faecium*, in silico epitope mapping has identified conserved surface proteins, including the zinc-dependent lipoprotein AdcA, as dominant immunogenic targets. The hyper-thermostable multi-presentation antigen Sc(EH)3, designed based on predicted epitopes of AdcA’s zinc-binding domain, induces opsonic antibodies conferring protection against methicillin-resistant *S. aureus* (MRSA) and vancomycin-resistant *E. faecium* (VREfm) strains. This exemplifies how structure-based epitope prediction supports vaccine candidate optimization. Additionally, reverse vaccinology and machine learning tools like VacSol-ML utilize bulk and single-cell RNA sequencing to prioritize candidate antigens exhibiting high expression and immunogenicity with minimal off-target effects [15,16,92,93].

In Gram-negative pathogens, computational analyses have revealed conserved outer membrane proteins and siderophore receptors as promising vaccine targets. For *K. pneumoniae*, outer membrane proteins such as OmpK36, siderophore receptors, and capsule-associated proteins have been identified through integrated transcriptomic and immunopeptidomics data, highlighting antigens abundantly expressed during infection and presented on host MHC molecules. Machine learning models predict epitopes with strong binding affinity across diverse HLA alleles, facilitating broad population coverage. In *A. baumannii*, conserved proteins like OmpA and BauA, associated with adhesion and iron acquisition, have been validated as immunogenic through proteogenomic workflows. Multi-epitope vaccine constructs designed from these epitopes address antigenic diversity to enhance protective efficacy. Similarly, in *P. aeruginosa*, conserved outer membrane proteins (OprF, OprI), type III secretion system components, and siderophore receptors have been prioritized as vaccine candidates. Immunoinformatics approaches combining pathogen transcriptomics and host HLA allele frequency data identify epitopes eliciting robust immune responses, while structural modeling assists in selecting epitopes with optimal MHC binding and T cell receptor engagement [21,40,79,94,95,96,97,98,99,100].

Although less extensively characterized, *Enterobacter* species have been examined using machine learning frameworks that integrate genomic and proteomic data to discover conserved outer membrane proteins and virulence factors as candidate antigens. Reverse vaccinology has identified epitopes with high predicted immunogenicity and low human homology, providing a foundation for vaccine development against these emerging multidrug-resistant pathogens [70,101,102,103].

Despite these advances, computational antigen discovery faces challenges such as pathogen population heterogeneity, antigenic variation, and the complex host–pathogen interplay. Accurate immunogenicity prediction requires comprehensive data profiling and experimental validation to distinguish pathogen-specific antigens from human homologs, thereby minimizing autoimmune risks. Emerging AI-based methods trained on extensive experimentally validated datasets hold promise for improving prediction accuracy. The integration of computational antigen discovery with innovative vaccine platforms like mRNA vaccines and outer membrane vesicles (OMVs) represents a promising strategy to develop next-generation vaccines against ESKAPE pathogens [15,104,105].

So, the application of computational antigen design, leveraging multi-omics data and machine learning, provides a rational framework for identifying conserved, immunogenic epitopes with broad population coverage and minimal cross-reactivity. This approach supports the development of multi-epitope and cross-protective vaccines capable of overcoming antigenic diversity and immune evasion. Combining in silico epitope prediction with established vaccine platforms such as mRNA and OMVs is poised to revolutionize prevention strategies against multidrug-resistant ESKAPE infections [13,15,20].

Across recent in silico vaccine-discovery studies targeting the *Enterobacter* spp., several promising antigen candidates have consistently emerged, most notably outer-membrane proteins (OMPs), periplasmic transporters, and secreted virulence-associated proteins that are conserved, non-homologous to humans, and strongly antigenic. The *Subtractive Genomic Analysis* approach applied in the first study used a classical reverse vaccinology pipeline involving BPGA for pangenome analysis, PSORTb for subcellular localization, VFDB for virulence mapping, BLASTp to exclude host-homologs, and VaxiJen for antigenicity prediction. This pointed to conserved OMPs—such as TonB-dependent receptors, OmpA-like proteins, and transport-related membrane proteins—as high-value candidates for vaccine development. Another study used subtractive genomics by generating a full multi-epitope vaccine construct from carefully selected surface-exposed proteins. Its pipeline integrated PSORTb, CELLO, and SignalP for secretome/surfaceome filtering; IEDB tools (BepiPred, NetMHCpan, NetMHCIIpan) for B-cell and T-cell epitope prediction; VaxiJen, AllerTOP, and ToxinPred for safety profiling; and structural refinement via I-TASSER, GalaxyRefine, and molecular docking with ClusPro and HADDOCK. Immune simulation using C-ImmSim suggested that epitopes derived from OmpA-like proteins, siderophore receptors, and membrane enzymes could elicit robust humoral and cellular responses. Similarly, the *Computer-Aided Multi-Epitope Vaccine Design against Enterobacter* spp. study applied a streamlined but comparable computational workflow. It used ExPASy ProtParam for physicochemical filtering, PSORTb and TMHMM for predicting membrane localization, IEDB for epitope selection, VaxiJen and AllergenFP for immunogenicity and allergenicity screening, and AlphaFold2/I-TASSER for structural modeling. Docking with AutoDock Vina and immune simulation via C-ImmSim highlighted multi-epitope constructs built from OmpA, LptD, and siderophore receptors as some of the most promising candidates. Collectively, these three studies converge on a highly consistent finding: conserved outer-membrane proteins of *Enterobacter* spp. remain the strongest computationally predicted vaccine targets, and multi-epitope designs built from these antigens show favorable immunogenic profiles across all applied pipelines [41,69,70,106].

## 5. Vaccine Landscape Across the ESKAPE Pathogens

**Figure 4:**
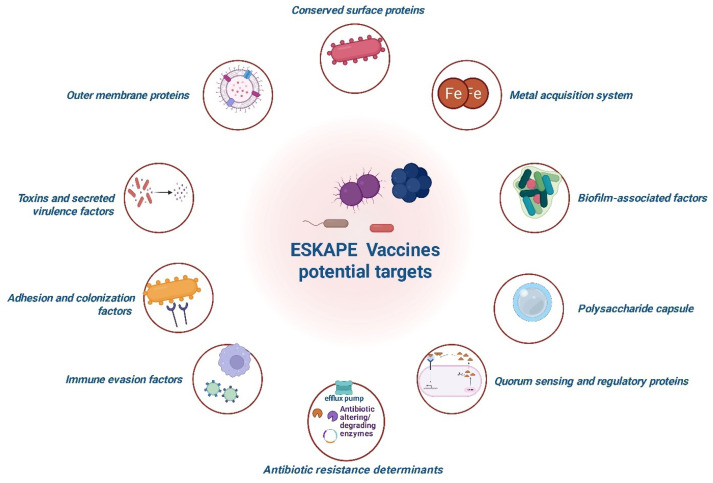
Potential vaccine targets against ESKAPE pathogens. The targets are categorized based on their roles in bacterial pathogenesis, virulence, and survival. These include conserved surface proteins that are broadly expressed among clinical isolates and capable of eliciting cross-protective immune responses; metal acquisition systems, such as siderophore-mediated iron uptake, which are essential for bacterial growth in the host environment; and biofilm-associated factors that facilitate persistent infections and protect bacteria from host immunity and antibiotics. Additional targets comprise the polysaccharide capsule, a major virulence determinant that shields pathogens from phagocytosis; quorum sensing and regulatory proteins that modulate gene expression related to virulence and resistance; and antibiotic resistance determinants, including efflux pumps and antibiotic-degrading enzymes. Other key targets are immune evasion factors that interfere with host immune recognition and clearance; adhesion and colonization factors that mediate attachment to host tissues; secreted toxins and virulence factors that directly damage host cells; and outer membrane proteins involved in structural integrity, nutrient acquisition, and immune interaction. Collectively, these components represent promising candidates for the development of effective vaccines to combat infections caused by multidrug-resistant ESKAPE pathogens. (Created with BioRender.com).

Several promising antigenic targets have been identified across the ESKAPE pathogens, as illustrated in Figure 4, which will be detailed as follows. Examples of vaccine candidates against each pathogen are summarized in Table 1, including their composition, immunogenicity, and stage of development.

### 5.1. Enterococcus faecium Vaccine Candidates

Vaccine strategies against *Enterococcus faecium* focus on highly conserved surface-anchored proteins critical for adhesion, metal uptake, and biofilm formation. One promising candidate is the zinc-binding lipoprotein AdcA, which mediates zinc acquisition, an essential function for bacterial survival and virulence. Immunization with recombinant AdcA or a computationally designed epitope-based antigen (Sc(EH)3) elicits strong opsonophagocytic antibodies and protects mice in systemic infection models. Importantly, these antibodies cross-react with *Staphylococcus aureus*, supporting the idea of a multi-antigen vaccine effective across Gram-positive ESKAPE pathogens [123].

Other major surface-exposed antigens under investigation include Enterococcal surface protein (Esp), Secreted antigen A (SagA), and MSCRAMM adhesins. Esp promotes adherence to host tissues and fosters biofilm formation, which correlates with persistence in vivo and contributes to virulence in endocarditis models. Despite its importance, Esp exhibits antigenic variability and partial redundancy with other adhesins, which limits its utility as a standalone vaccine target [123,124,125,126,127]. SagA aids in immune evasion, allowing *E. faecium* to survive against host defenses by activating TLR2 on FOXP3+ regulatory T cells, promoting immunologic tolerance and enhancing the colonization of commensal bacteria [128,129].

Biofilm formation enhances the survival of ESKAPE pathogens, For *E. faecium* it protect it from antibody-mediated immune responses, creating a VRE strain that complicates therapeutic interventions [130,131]. Biofilm formation is further supported by pili structures, notably the endocarditis and biofilm-associated pilus (Ebp) [132,133], which are crucial for colonization, persistence, and disease progression, especially in endocarditis. Other surface proteins, including PrgB, sense environmental cues and enhance adaptation and virulence [134,135]. Given the challenges of vaccine efficacy against biofilms, researchers are exploring multi-antigen vaccine strategies for *Enterococcus*, combining surface adhesins such as Esp, pilus proteins (e.g., EbpA), and other conserved surface components. These approaches aim to broaden immunogenicity and functional protection, though to date, preclinical work has mainly focused on individual antigens or cocktails that do not yet include all three classes together [65,136].

Polysaccharide capsules are less studied compared to Gram-negative ESKAPE pathogens, and the occurrence of capsular *E. faecium* polysaccharides as potential vaccine candidates is not well understood at this stage. Nevertheless, biofilm proteins and extracellular matrix components are emerging as supplementary targets for the prevention and treatment of chronic infections [33,92,137,138].

*E. faecium* has peptide-based QS mechanisms common to Gram-positive bacteria, which are mainly mediated through two-component systems and gene regulators, including RNPP family proteins (Rap, NprP, PlcR, PrgX) and Rgg family transcriptional regulators. These systems control conjugative plasmid transfer, biofilm growth and the expression of virulence determinants. QS in *E. faecium* regulates antibiotic resistance phenotypes by regulating efflux pumps and biofilm-related genes, and this QS mechanism may contribute to persistence in nosocomial environments. Vaccination or small-molecule inhibitors against peptide autoinducers or their corresponding receptors may block these pathways, thereby limiting both virulence and resistance [139,140].

*E. faecium* primarily acquires vancomycin resistance through horizontal gene transfer of the *vanA* and *vanB* gene clusters, often carried on mobile elements like plasmids and transposons. These genes are induced by vancomycin exposure, enabling rapid bacterial adaptation and proliferation under antibiotic pressure. mRNA vaccines targeting *vanA* and *vanB* deliver mRNAs encoding segments of these genes to host cells can be used to neutralize resistant *E. faecalis* strains and restore vancomycin efficacy [141,142].

Ace (adhesin to collagen of Enterococcus) is a critical surface protein that aids in the adhesion of *E. faecalis* to collagen, a major component of the host extracellular matrix. Ace, approximately 88 kDa in size, facilitates the colonization of host tissues, contributing to the pathogenicity of the bacterium. By inhibiting the binding of Ace to host cells, the damage caused by *E. faecium* can be reduced [143]. Studies have shown that immunizing rats with recombinant Ace (rAce) decreases their susceptibility to OG1RF infection, highlighting the potential of Ace as a vaccine target against *E. faecalis*. An mRNA vaccine targeting Ace introduces mRNA encoding the Ace antigen into host cells, promoting the production of the Ace protein [132,143].

### 5.2. Staphylococcus aureus Vaccine Candidates

In case of *S. aureus* Vaccine strategies have largely focused on surface-exposed proteins and adhesins that play a major role in bacterial virulence, colonization, and immune evasion. Of the surface antigens, the iron-regulated surface determinants (Isd) such as IsdA, IsdB, SdrD, and SdrE have been among those most thoroughly studied and have shown promising protective efficacy in preclinical models [144]. The proteins are engaged in iron acquisition and host tissue attachment and were also shown to be capable of promoting the binding of heme (IsdB and IsdA) and hemoglobin (IsdB) that subsequently goes on to scavenge iron, a critical requirement for bacterial survival in a host. Both SdrD and SdrE are MSCRAMMs of the serine-aspartate repeat CW family, involved in attachment to host extracellular matrix constituents. Immunization with four single antigens resulted in a 3–4 log decrease in bacterial load in murine kidney infection compared to controls and combined vaccination provided enhanced protection. Significantly, antibodies against these proteins are able to mediate opsonophagocytic killing by human neutrophils, a key correlate of protective immunity [145,146,147,148,149,150,151]. However, vaccination with the V710 vaccine (iron surface determinant B, ISdB) was associated with a notable increase in multiple organ failure incidence and mortality among patients undergoing cardiothoracic surgery who developed *S. aureus* infections [113]. Moreover, the zinc-binding lipoprotein AdcA, homologous to Isd components, plays a critical role in metal uptake and has been rationally designed into vaccine antigens such as Sc(EH)3, the study reported that antibodies raised against Sc(EH)3 mediate opsonic killing of a wide-spectrum of Gram-positive pathogens, including VREfm and MRSA, and confer protection both in passive and active immunization models [92,145].

Biofilm-associated targets include polysaccharides such as poly-N-acetylglucosamine (PNAG), extracellular DNA (eDNA), and surface proteins like Bap, SasC, and fibronectin-binding proteins (FnBPA, FnBPB). These components promote adhesion, aggregation, and immune evasion, contributing to chronic infections and antibiotic resistance. Vaccines against PNAG and interventions targeting quorum-sensing (QS) pathways such as the Agr system have demonstrated efficacy in limiting biofilm formation and toxin production, though allele diversity complicates development [152,153,154,155,156].

Multicomponent protein vaccines have advanced to clinical stages. The four-antigen *Staphylococcus aureus* vaccine (SA4Ag) is composed of capsular polysaccharide conjugates of serotypes CP5 and CP8 (to CRM_197_), recombinant clumping factor A (ClfA), and recombinant manganese transporter protein C (MntC) [108]. In preclinical animal models—including mouse bacteremia, deep-tissue infection, pyelonephritis, and rat endocarditis—SA4Ag vaccination significantly reduced bacterial burden and in some cases provided sterilizing immunity [109]. In a Phase 1/2 trial in healthy adults (18–64 years) (NCT01364571), SA4Ag was well tolerated and induced strong functional immune responses: by Day 29, opsonophagocytic activity (OPA) titers increased ~69-fold for CP5 and ~29-fold for CP8; antigen-specific competitive Luminex immunoassay (cLIA) titers increased ~20-fold for MntC and ~12-fold for ClfA [108]. In older adults (65–85 years) (NCT01643941), a single dose elicited >50-fold OPA increase for CP5 and >20-fold for CP8, with acceptable safety [157]. In Japanese adults (20–85 years) (NCT02492958), >85% of recipients achieved predefined thresholds for all four antigens by Day 29, and antibody titers remained above baseline for 12 months [158]. In the Phase 2b/3 STRIVE trial (NCT02388165) in patients undergoing elective open posterior spinal fusion surgery, SA4Ag showed 0% efficacy versus placebo in preventing postoperative *S. aureus* bloodstream infection or deep surgical site/organ-space infection by Day 90, despite robust antibody responses [107]. Analysis of the STRIVE trial and other clinical studies indicates that, although SA4Ag elicited strong functional antibody responses against all four antigens, these responses did not translate into protection against invasive *S. aureus* infections in surgical patients [110]. Reviews of SA4Ag and other multivalent *S. aureus* vaccines, including V710, highlight several factors such as the complexity of *S. aureus* pathogenesis and its multiple redundant virulence mechanisms, the limitations of animal models in predicting human immune protection, and challenges in achieving sufficient immune coverage with selected antigens alone [109,144,159].

Another example is the recombinant five-antigen *Staphylococcus aureus* vaccine (rFSAV), which is a multivalent recombinant protein vaccine combining five conserved *S. aureus* antigens (α-hemolysin/Hla, staphylococcal enterotoxin B/SEB, SpA, IsdB fragment, and MntC). In animal models, it provided broad protection; rFSAV progressed into human trials and Phase 1a/1b clinical testing (NCT02804711, NCT03966040) that showed it to be safe and immunogenic (robust antigen-specific antibody and cellular responses). See the preclinical/early clinical reports for experimental details and trial IDs [160].

Other approaches include mRNA-LNP cocktails encoding multiple virulence factors (MntC, SEB, Hla, FnBPA, IsdB), inducing protective immunity in murine challenge models and reducing morbidity/mortality following *S. aureus* (including MRSA) challenge [161]. Another vaccine is the Intranasal nanoparticle vaccines in which Targeted intranasal delivery of *S. aureus* antigen(s) using antigen-loaded nanoparticles elicited mucosal immunity (IgA) and protected mice from subsequent systemic *S. aureus* challenge in experimental models [162]. Additionally, the Extracellular vesicle (EV)-coated nanovaccines Are which are engineered nanoparticles coated with *S. aureus* extracellular vesicles (EVs) to present multiple bacterial antigens in a particulate vaccine format. In murine models the EV-coated nanovaccine induced strong CD4+ and CD8+ responses and protected against drug-resistant *S. aureus* skin infection; adoptive/functional assays supported both humoral and cellular mechanisms [163]. Furthermore, the Sta-V5 prophylactic multivalent subunit vaccine comprises five conserved *Staphylococcus aureus* antigens: AdsA (adenosine synthase A), EsxA, EsxB, PmtA and PmtC [164]. In mouse sepsis/bacteremia models, Sta-V5 elicited opsonic antibodies and significantly improved survival versus single-antigen vaccines [164]. In addition, recent experimental work using PLGA/PEG nanoparticles loaded with recombinant *S. aureus* antigen rEsxB (and rEsxA in some designs) reported 100% protection in mouse infection models following subcutaneous immunization (numeric survival and CFU reductions reported [165].

Currently, a multivalent toxoid vaccine candidate, LBT-SA7, designed to prevent skin and soft tissue infections caused by *S. aureus*, is in Phase 1 clinical trials with initial results expected in late 2025. This vaccine includes weakened forms of multiple *S. aureus* toxins to neutralize their harmful effects and represents a novel approach to vaccine development against this pathogen, which is a major cause of global morbidity and mortality, with rising antibiotic resistance [166,167].

The use of extracellular vesicles (EVs) derived from *Staphylococcus aureus* has emerged as a promising multivalent vaccine strategy, owing to their ability to present a native cargo of surface and secreted antigens in a single particulate form. In one pivotal study, mice immunized with *S. aureus* EVs developed strong Th1-polarized immune responses (notably IFN-γ production), and adoptive transfer of T cells—but not sera—conferred protection against both lethal airway challenge and pneumonia models, indicating T-cell-dependent protection [168]. A subsequent investigation demonstrated that EVs purified from a detoxified strain of *S. aureus* retained antigenicity and were protective in a murine sepsis model, further underscoring the potential of EVs as vaccine platforms [169]. While translation to human use will require careful attention to EV heterogeneity, safety (e.g., toxin carry-over) and antigen mapping, these preclinical studies provide strong in vivo proof-of-concept that *S. aureus* EVs can confer protection against severe infection.

Epitope-based vaccine development against *Staphylococcus aureus* has shown encouraging preclinical outcomes supported by multiple experimental studies. One study demonstrated that vaccines composed of multiple B-cell epitopes derived from staphylococcal enterotoxin B (SEB) induced high IgG titers, mediated opsonophagocytic killing, and significantly improved survival in murine bacteremia models [170]. Similarly, MntC-specific B-cell epitope vaccines (MntC113–136-KLH, MntC209–232-KLH, and MntC263–286-KLH) generated strong antibody responses and conferred protection in mouse infection models [171]. In a recent study, seven immunodominant epitopes identified during clinical evaluation of a five-antigen vaccine (rFSAV) demonstrated that monoclonal antibodies against individual epitopes conferred partial protection in murine bacteremia, whereas combinations produced markedly improved survival [75]. In vivo protective efficacy has also been reported for linear B-cell epitopes from GapC and from fibronectin-binding protein A (FnBPA) [172,173,174]. Additionally, the TRAP-derived Als3 T-cell epitope vaccine (ATT) provided significant protection against lethal *S. aureus* challenge and induced strong Th1/Th17 responses [175], and the multi-epitope peptide MAP27, containing four peptidoglycan epitopes, reduced bacterial loads in organs and improved survival in murine bacteremia [72]. Complete protection in mouse sepsis models has also been reported from the CgoX-D epitope vaccine [74]. However, not all multi-epitope vaccines have succeeded: a formulation incorporating epitopes from eight virulence factors failed to induce antigen-recognizing antibodies and did not protect mice against infection, illustrating the complexity of epitope selection and immune correlates of protection [176].

### 5.3. Klebsiella pneumoniae Vaccine Candidates

*K. pneumoniae* synthesizes multiple siderophores—enterobactin, aerobactin, yersiniabactin, and salmochelin—which enable high-affinity scavenging of ferric iron from the host. These siderophores are recognized by specific outer-membrane receptors that mediate their uptake. Because these receptors are surface-exposed and immunogenic, they represent attractive vaccine targets. Experimental vaccines based on siderophore receptor antigens have demonstrated immunoprotective efficacy in animal models, although widespread antigenic variation and functional redundancy among siderophore systems remain major barriers to universal vaccine design [177,178,179,180,181]. To expand the scope of *K. pneumoniae* vaccine development, researchers are exploring additional targets such as MagA (mucoviscosity-associated gene A), which is involved in capsule production and virulence [182]. LPS, a pivotal component of the immune response, is also considered a potential vaccine target. Furthermore, adhesive organelles such as Type 1 and Type 3 fimbriae, as well as siderophores such as EntB and EntC, are being investigated owing to their roles in tissue adhesion and iron chelation [183,184,185,186,187,188,189].

Biofilm formation in *Klebsiella pneumoniae* heavily depends on type 3 fimbrial adhesins, while capsular polysaccharides are major surface components influencing adhesion. Vaccination against fimbrial proteins, such as MrkA (type 3) and FimA (type 1), has been shown to reduce in vitro biofilm formation and enhance bacterial clearance in animal models. Intracellular cyclic di-GMP signaling, regulated by diguanylate cyclases such as DgcG, modulates biofilm formation and fimbrial expression, indicating that c-di-GMP pathways control structural adhesins. Furthermore, MrkH, a c-di-GMP-dependent transcriptional activator, directly regulates the expression of type 3 fimbriae, linking intracellular signaling to biofilm development. These findings suggest that components of the c-di-GMP/MrkH pathway represent promising targets for preventing biofilm maturation and enhancing antimicrobial susceptibility [185,190,191,192,193].

Vaccines against *Klebsiella pneumoniae* have primarily focused on polysaccharide–protein conjugates targeting the bacterium’s capsular polysaccharides (CPSs), with serotypes such as K1 and K2 being the most common targets [189,194]. Experimental studies in mice have demonstrated that K1 and K2 CPS–CRM197 conjugate vaccines induce strong antigen-specific IgG responses and confer significant protection against lethal challenges with homologous strains [189,194]. Despite this success, the high diversity of *K. pneumoniae* capsular types presents a major challenge for broad-spectrum vaccine development, as strains express over 70 distinct K antigens globally [195,196].

Additional approaches have investigated O-antigen (LPS) and fimbrial targets: O1 O-antigen glycoconjugates and VLP-based vaccines elicited robust humoral responses and protected mice against lethal challenge [196,197], whereas type 1 and type 3 fimbrial adhesins contribute to biofilm formation and may serve as complementary vaccine antigens [198]. Live-attenuated KbvR mutant strains, which alter capsular and fimbrial expression, also elicited strong humoral immunity and complete protection in mouse models [198]. Collectively, these findings provide experimental evidence that CPS-, LPS-, fimbrial-, and live-attenuated-based vaccines are immunogenic and protective in animal models, but capsular heterogeneity remains a key barrier to developing a universally effective *K. pneumoniae* vaccine.

*Klebsiella pneumoniae* vaccine research has demonstrated promising results using conserved outer-membrane proteins (OMPs). In a murine infection model, immunization with recombinant OmpK36 (and OmpK17 or a fusion protein) elicited IgG responses and provided up to 60% survival after a lethal bacterial challenge [199]. DNA vaccination using plasmids expressing OmpA or OmpK36 similarly produced antigen-specific IgG, induced mixed Th1/Th2 cytokines, enhanced opsonophagocytic activity, and protected mice against lethal *K. pneumoniae* infection [95]. In addition, a recombinant multi-epitope subunit vaccine (r-AK36), composed of epitopes from OmpA and OmpK36, induced strong humoral responses, inhibited biofilm formation in vitro, and conferred ~80% survival in mice challenged with a virulent strain [79]. Furthermore, more recent proteomics and immunologic screening identified five highly conserved OMPs in *K. pneumoniae*; three of these (named Kpn_Omp001, Omp002, and Omp005) induced protective immunity via IFN-γ, IL-4 and IL-17A responses in mouse challenge models, supporting their potential as broadly effective, serotype-independent vaccine antigens [37].

Additionally, another study developed a multi-epitope vaccine, mHla-EpiVac, incorporating FepA, OmpA, and OmpW antigens on an alpha-hemolysin mutant (mHla) heptamer platform, which activated and matured bone marrow-derived dendritic cells (BMDCs), elicited robust humoral and cellular immune responses, and conferred protective efficacy in a mouse acute pneumonia model [80]. Fimbrial adhesin MrkD has also been experimentally investigated: three Th-cell epitopes (M221–235, M175–189, M264–278) induced IL-4 and IFN-γ production and evoked CD4+ T-cell responses in immunized mice [200].

### 5.4. Acinetobacter baumannii Vaccine Candidates

*Acinetobacter baumannii* relies on siderophore-mediated iron acquisition, and BauA, the outer-membrane receptor for acinetobactin, has been validated as a vaccine antigen. Immunization with BauA (or its loop-derived fragments) elicits specific IgG responses and partially protects mice in sepsis challenge models [201,202]. Combining BauA with other antigens, such as HemTR, enhances protection—the HemTR-BauA combination reduced bacterial load and delayed mortality in mice [203]. Similarly, a BauA + Bap (biofilm-associated protein) combination provides synergistic immunoprotection: mice receiving the combination vaccine developed significantly higher IgG titers than single-antigen groups and showed markedly reduced bacterial loads in spleen, liver, and lungs [204]. A two-protein vaccine composed of BauA and Omp34 achieved full survival in actively immunized mice and ~86% survival upon passive transfer of immune serum [205]. Outer-membrane vesicle (OMV)-based vaccines derived from *A. baumannii* induce robust humoral responses and protect mice in both pneumonia and sepsis models [206]. A novel nanoparticle–OMV hybrid vaccine (gold nanoparticles coated with *A. baumannii* OMVs) has further shown strong IgG induction, opsonophagocytic activity, and protection in mice and rabbits, demonstrating an advanced and promising platform for vaccine development [207].

*Acinetobacter baumannii* is a multidrug-resistant nosocomial pathogen whose biofilm-forming capacity, mediated by outer membrane proteins (OMPs), pili, and exopolysaccharides, contributes to persistence in hospital environments and resistance to antibiotics. Several surface antigens have been experimentally validated as vaccine candidates in animal models [208,209,210,211,212,213,214]. OmpA, a highly conserved β-barrel protein, functions as an adhesin and promotes biofilm formation. Immunization with recombinant OmpA, DNA vaccines, or intranasal formulations elicits strong humoral and cellular immune responses, reduces bacterial loads, and increases survival in murine infection models [205,215,216,217]. Omp33-36 (Omp34), another porin involved in host–cell interaction and virulence, has demonstrated protective efficacy when administered alone or in combination with the siderophore receptor BauA, significantly reducing bacterial burden and improving survival in sepsis and pneumonia models [98,201,205,213,218]. Similarly, immunization with Omp22 confers protection against lethal challenge and enhances opsonophagocytic killing, with fusion constructs or nanoparticle-based delivery further improving efficacy [212,219,220]. OmpW and the trimeric autotransporter Ata have also been shown to induce protective antibody responses and reduce organ bacterial loads in murine infection models [206,221,222]. Additionally, outer membrane vesicle (OMV)-based vaccines presenting multiple native antigens have demonstrated robust immunogenicity and protection in both active and passive immunization studies [214]. Furthermore, immunization with a DcaP-like outer membrane protein together with AbOmpA induced strong IgG responses and provided protection in a sepsis model, reducing bacterial burden in lung and spleen [223]. Collectively, these findings highlight the potential of OMP-based and OMV-based formulations as promising strategies for the development of effective vaccines against multidrug-resistant *A. baumannii*.

In parallel, reverse vaccinology and immunoinformatic approaches have expanded the repertoire of potential vaccine antigens. For example, a comprehensive in silico screening across multiple *A. baumannii* strains identified membrane-exposed proteins such as CarO, OmpH, LptE, and FimF as conserved, antigenic, and potentially non-allergenic targets [99,224,225,226,227]. While these candidates have not yet been validated in experimental infection models, their high conservation and favorable antigenic profiles make them highly promising for future vaccine development.

### 5.5. Pseudomonas aeruginosa Vaccine Candidates

*P. aeruginosa* synthesizes siderophore-pyoverdine, which is essential for iron uptake and biofilm development. The uptake and virulence factor, pyoverdine receptor FpvA is essential. Immunization with the FpvA-KLH vaccine decreased the bacterial burden and lung edema after *P. aeruginosa* challenge. Pyoverdine or FpvA mutants’ virulence and in vivo pathogenic biofilm formation are decreased. Vaccines targeting pyoverdine biosynthesis or receptor proteins have been shown to protect in animal models, suggesting their potential for vaccine development [228,229,230,231,232,233,234].

*Pseudomonas aeruginosa* biofilms are a major cause of chronic and persistent infections, particularly in patients with cystic fibrosis. Biofilm formation in *P. aeruginosa* is supported by a complex extracellular matrix composed primarily of the exopolysaccharides alginate, Pel, and Psl, together with extracellular DNA (eDNA) and matrix-associated proteins. Experimental studies have demonstrated that alginate plays a central role in biofilm integrity and resistance to host immune responses [235,236,237,238,239,240,241]. Several experimental studies have demonstrated the protective potential of alginate-based conjugate vaccines against *Pseudomonas aeruginosa* in murine models. For example, alginate–OMV conjugates elicited robust humoral immune responses and significantly reduced bacterial burden in the lungs of vaccinated mice following intranasal challenge, highlighting the ability of outer membrane vesicles to enhance antigen presentation and immunogenicity [242]. Similarly, a polymannuronic acid (PMA)–flagellin conjugate, where PMA represents a structural component of alginate, induced high titers of anti-PMA antibodies in mice and conferred protection in an experimental lung infection model, as evidenced by decreased bacterial load and improved survival [243]. In addition, an alginate–diphtheria toxoid (DT) conjugate vaccine was shown to be highly immunogenic, eliciting strong IgG and IgM responses in mice, supporting the feasibility of carrier-protein conjugates to enhance the immunogenicity of polysaccharide antigens [244]. Collectively, these studies confirm that alginate-based conjugate vaccines can stimulate protective immunity against *P. aeruginosa* and reduce infection severity in vivo, providing a promising approach for vaccine development against chronic and biofilm-associated infections.

*Pseudomonas aeruginosa* outer membrane vesicle (OMV) vaccines have demonstrated protective efficacy in preclinical models: for example, immunization of mice with PA-derived OMVs formulated with alum reduced bacterial colonization, dampened inflammatory cytokine production, and significantly improved survival in a lethal lung-infection challenge [115]. for instance, OMVs conjugated to diphtheria toxoid (PA-OMVs-DT) elicited high IgG titers and reduced bacterial burden and inflammation in a murine burn infection model [39]. Recombinant outer membrane proteins, notably a hybrid OprF–OprI (Met-Ala-(His)_6_-OprF_190–342_–OprI_21–83_), have also been shown to elicit strong opsonophagocytic and complement-activating antibody responses. Subcutaneous vaccination of BALB/c mice with OprF/I (with or without flagellin B) significantly prolonged survival, reduced bacteremia and diminished lung pathology upon challenge with either mucoid or nonmucoid strains [245]. The same OprF/I fusion antigen (IC43) has advanced into human clinical trials: in a randomized, placebo-controlled phase I study, healthy adults receiving two doses 7 days apart developed robust OprF/I-specific IgG responses and functional opsonophagocytic activity, with an acceptable safety profile [116]. Additionally, a recombinant triple fusion vaccine combining OprF, OprI, and PcrV provided enhanced protection in a burn-mouse model, significantly increasing survival and reducing bacterial load in multiple organs [117]. Finally, targeting the type III secretion system (T3SS) tip protein PcrV has also shown promise: passive administration of rabbit anti-PcrV F(ab′)2 fragments conferred full protection in a mouse sepsis model, and reduced lung injury and bacteremia in a rabbit model of ventilator-associated Pseudomonas pneumonia [246].Taken together, these studies provide strong in vivo proof-of-concept for multiple antigen strategies (OMVs, surface OMPs, T3SS targets) against *P. aeruginosa*.

While multiple *Pseudomonas aeruginosa* vaccine strategies have shown promise in preclinical models, translation to clinical benefit has been challenging. For instance, the recombinant OprF/I fusion vaccine IC43 demonstrated excellent immunogenicity in mechanically ventilated ICU patients, but a large phase 2/3 trial found no significant reduction in 28-day all-cause mortality compared to placebo, despite robust antibody responses [247]. Similarly, KB001-A, a PEGylated anti-PcrV monoclonal antibody fragment targeting the T3SS tip protein, was safe and well-tolerated in a cystic fibrosis cohort, but failed to extend the time to antibiotic use in a 16-week study—likely due to low expression of T3SS proteins in chronically colonized CF airways [248]. These failures underscore the complexity of developing effective vaccines for *P. aeruginosa*, particularly in chronically infected or critically ill patient populations.

### 5.6. Enterobacter *spp.* Vaccine Candidates

Experimental vaccine-prototype investigations in *Enterobacter* spp. remain rare, but a few proof-of-concept studies highlight feasibility. A study group generated *Enterobacter aerogenes* “bacterial ghosts” using a sponge-like reduced protocol (SLRP), and immunized mice (three doses) followed by live challenge. Vaccinated mice showed significantly higher antigen-specific IgG, IgA, and IgM, elevated cytokines (TNF-α and IL-10), reduced bacterial burden in liver and spleen, and improved histopathology, with cross-reactive serum responses to other Enterobacteriaceae [249]. In a separate study, researchers used formaldehyde-inactivated multidrug-resistant *Enterobacter cloacae* to immunize Swiss albino mice intradermally over three doses; after intraperitoneal lethal challenge, 100% of immunized mice survived, and sera from these mice demonstrated strong bactericidal activity and high IgG binding by ELISA [250]. In addition to these protection studies, another study characterized outer membrane vesicles (OMVs) from *Enterobacter cloacae* ATCC 13047 by proteomics, identifying numerous membrane-bound and transporter proteins—data that could underlie future OMV-based vaccine designs [251].

## 6. Insights from Failed Single-Target and Shifting Toward Cross-Genus Target Approaches

Clinical attempts to prevent infections caused by *Staphylococcus aureus* and *Pseudomonas aeruginosa* by targeting single virulence factors have largely been unsuccessful. Vaccines and monoclonal antibodies against ClfA, a fibrinogen-binding adhesin of *S. aureus*, induced high antibody titers and functional opsonophagocytic activity in preclinical models, but failed to confer meaningful protection in humans. This failure has been attributed to multiple factors: *S. aureus* expresses a diverse repertoire of adhesins and virulence factors, rendering single-target strategies insufficient; humans already harbor circulating antibodies against ClfA and other surface antigens yet remain susceptible to infection; and correlates of protective immunity remain poorly defined, with many animal models not fully recapitulating human immune evasion mechanisms [252].

Similarly, the anti-PcrV Fab fragment KB001, designed to neutralize the tip protein of the Type III secretion system in *P. aeruginosa*, was well tolerated and detectable in airway secretions, yet it did not achieve clinical benefit in cystic fibrosis patients chronically colonized with the bacterium. Mechanistic explanations for this failure include the transient expression of PcrV during infection, the multifactorial pathogenesis of chronic lung disease—including biofilm formation and mucoid phenotypes—and suboptimal tissue penetration of the antibody [119,253].

These experiences highlight that effective prophylaxis against ESKAPE pathogens likely requires multivalent strategies targeting multiple antigens and virulence pathways, rather than relying on a single target. Preclinical studies support this approach: for *S. aureus*, combinations of antigens that induce both humoral and cellular immunity, including Th1/Th17 responses, are recommended; for *P. aeruginosa*, vaccines incorporating PcrV with additional secretion system proteins or outer membrane components have demonstrated higher survival and immune protection in animal models [254,255]. Additionally, timing and context of intervention are critical: early targeting of virulence factors, ensuring sufficient antibody or effector penetration into infected tissues, and combining immunization with adjunctive therapies such as antibiotics or biofilm disruptors may further improve efficacy. Overall, these findings emphasize that while antigen selection is necessary, a more integrated, multi-target, multi-mechanism approach is likely required for successful vaccine development against multidrug-resistant pathogens.

## 7. Structural Basis and Mechanistic Rationale for Cross-Reactive Antigen Targeting

Cross-reactivity of vaccine-elicited responses is best achieved when antibodies or T cells target conserved, structurally-critical epitopes that are present in multiple strains or species. For example, a study group reported that a single human monoclonal antibody neutralized α-hemolysin and four bi-component leukocidins of *Staphylococcus aureus* by binding a conformational epitope conserved among the toxins despite low overall sequence identity [256]. Similarly, in the present work the antigens selected (e.g., AdcA, BauA, OmpA, OmpK36) share surface-exposed regions or ligand-binding pockets that are under strong functional constraint and thus conserved across strains. By targeting these “vulnerable sites” (metal-binding pockets, extracellular porin loops, siderophore receptor clefts), the induced antibodies are likely to bind homologous proteins from different strains or even species. The inclusion of multiple conserved antigens in a multivalent design further increases breadth of coverage by reducing the likelihood of escape via antigenic variation.

Additional structural basis for cross-reactivity is strongly supported by recent published work. For example, Kolla et al. mapped B-cell epitopes in the outer-membrane porin OmpL across Enterobacteriaceae and found ~82% sequence identity plus conserved extracellular loop architecture, demonstrating that antibodies targeting such loops could be broadly cross-reactive [257]. Similarly, Domínguez-Medina et al. showed that immunization with a porin from *Salmonella Typhimurium* elicited protection, but cross-protection was limited to *S. Enteritidis* due to subtle amino-acid differences plus shielding by O-antigen—emphasizing that even conserved epitopes must also be accessible in the strain context [258]. In the siderophore-receptor space, Ullah et al. performed epitope-conservancy analyses on FepA, maltoporin and OmpW families and found peptides that are highly conserved across species, supporting their vaccine candidacy for cross-species coverage. These studies reinforce the key design principle: target antigens whose structural, surface-exposed and functionally constrained domains are conserved across strains/species to maximize cross-reactive immunity, while also paying attention to expression and accessibility context [259].

Broad-spectrum (pan-ESKAPE) vaccine strategies aim to exploit structurally conserved, functionally constrained targets (metal-binding proteins, siderophore receptors, porin loops) that are shared across strains or even genera. Recent experimental work shows this approach can produce cross-protective immunity in preclinical models: a multipresenting AdcA-derived antigen (Sc(EH)_3_) elicited opsonophagocytic antibodies and protected against multiple Gram-positive pathogens in murine models, demonstrating the concept of a single engineered epitope conferring broad activity [92]. Similarly, hybrid-loop antigens derived from the siderophore receptor BauA produced bactericidal/opsonic responses and full protection in mice against *Acinetobacter baumannii*, illustrating that structurally conserved surface loops can be effective cross-protective subunits [260].

Platform and design advances further increase feasibility for broad-spectrum approaches. Outer-membrane vesicle (OMV)–based vaccines and engineered multivalent/epitope-display constructs enable presentation of multiple conserved antigens and have shown strong immunogenicity in preclinical studies; OMVs in particular are attractive for Gram-negative ESKAPE members because they naturally present outer-membrane proteins and associated glycans and can be bioengineered to enrich desirable antigens [62].

Recent literature highlights that achieving a fully universal vaccine targeting all six ESKAPE pathogens remains unlikely in the near term due to substantial biological and structural challenges. Key limitations include steric shielding of surface antigens by the lipopolysaccharide O-antigen and capsular polysaccharides in Gram-negative bacteria, which restrict antibody access to many outer-membrane proteins, as experimentally demonstrated in several immunization and structural studies. Additionally, high antigenic variability outside conserved functional domains and diverse immune-evasion strategies reduce the probability that a single antigen or vaccine formulation can provide broad protection across genera [19].

However, partially universal approaches leveraging conserved antigens have shown realistic promise. Conserved outer-membrane proteins such as OmpA and TolC, and adhesins such as Cna and Acm, play central roles in bacterial fitness, adherence, and antibiotic resistance, and exhibit conservation across multiple clinically relevant species including Acinetobacter, Klebsiella, and Enterobacter. Recent pan-genome reverse-vaccinology pipelines have successfully identified immunogenic epitopes within these proteins and validated their predicted structural accessibility and immune activation using molecular docking and immune simulation models [261].

Encouraging preclinical findings from multi-epitope constructs and outer-membrane-vesicle–based vaccines support the feasibility of broad-spectrum protection strategies targeting conserved surface antigens rather than pursuing a single fully universal vaccine [14]. Emerging platforms such as mRNA vaccines may further enhance antigen combination flexibility and rapid adaptation to antigenic variation [19]. Collectively, current evidence supports a practical trajectory toward species-focused, cross-genus vaccines built around conserved functional proteins, rather than a completely universal formulation across all ESKAPE pathogens.

A biologically realistic approach to broad-spectrum antibacterial vaccination is to divide the challenge into pan-Gram-positive and pan-Gram-negative vaccine strategies, rather than pursuing a single universal pan-ESKAPE vaccine. Gram-negative ESKAPE pathogens share conserved outer membrane proteins—such as BamA, LptD, and OmpA—enabling cross-reactive immunity. Indeed, vaccination with BamA from *A. baumannii* protects mice and generates antibodies that also bind *Klebsiella pneumoniae* and *E. coli* OMPs [262]. Similarly, *A. baumannii* OMVs and nanoparticle-OMV hybrids induce strong IgG and protective immunity in sepsis and pneumonia [206,207]. On the Gram-positive side, sortase A, pili subunits, and staphylococcal adhesins exhibit cross-species conservation across *Staphylococcus* and *Enterococcus*, and vaccination against these antigens provides protection in multiple murine infection models). Collectively, these findings support the feasibility of pan-Gram-positive and pan-Gram-negative vaccine platforms, which are scientifically more achievable than a universal pan-ESKAPE vaccine.

## 8. Conclusions and Future Perspectives

The development of effective vaccines against multidrug-resistant ESKAPE pathogens remains a critical global health priority. Recent advances emphasize the importance of inducing robust mucosal immunity, as most ESKAPE pathogens colonize mucosal surfaces such as the respiratory, gastrointestinal, and urinary tracts. Vaccines that stimulate mucosal immune responses-including secretory IgA and tissue-resident memory T cells-are essential to prevent colonization and transmission, yet most current vaccines primarily elicit systemic immunity. Enhancing mucosal protection requires continued research into targeted delivery systems and adjuvants that promote Th1 and Th17 responses, which are vital for clearing intracellular and extracellular bacteria [15,263,264,265].

Innovations in vaccine platforms, including mRNA technologies, outer membrane vesicles (OMVs), and nanoparticle-based delivery systems, offer promising avenues for improved antigen presentation and immune activation. However, challenges persist in selecting antigens that provide broad cross-strain protection amid extensive antigenic variability and immune evasion mechanisms characteristic of ESKAPE pathogens. The complexity of infections, including biofilm formation and chronic disease states, underscores the need for advanced preclinical models such as humanized immune systems, organoids, and microfluidic devices to better predict vaccine efficacy and safety [15,19].

Identification of immune correlates of protection remains an unmet need; discovering reliable biomarkers will facilitate candidate selection and streamline clinical trial design. Addressing antigenic diversity and immune escape will likely require integrated, multidisciplinary strategies combining conserved antigen discovery, multivalent vaccine formulations, and synergy with adjunctive therapies such as antibiotics, phage therapy, or microbiome modulation.

Reverse vaccinology and computational antigen prediction have accelerated the design of multivalent vaccines targeting conserved structures like surface adhesins, iron-uptake proteins, biofilm-associated antigens, and quorum sensing molecules [266]. Coupling these advances with state-of-the-art platforms will help overcome antigenic variability and immune evasion. Nonetheless, translating these innovations into clinically effective vaccines demands sustained interdisciplinary collaboration, improved preclinical models, and novel delivery and adjuvant systems that enhance immunogenicity and safety.

Furthermore, the translation of these vaccine platforms into real-world application requires navigating evolving regulatory frameworks and manufacturing challenges. Large-scale production of mRNA, OMV-based, and multivalent protein vaccines must comply with stringent quality-by-design and GMP standards, including control of antigen consistency, purity, endotoxin levels, and stability profiles. Regulatory agencies are increasingly developing platform-specific guidance—particularly for mRNA and vesicle-based vaccines—which mandates validated potency assays, robust characterization of delivery systems, and demonstration of batch-to-batch reproducibility. Additionally, scaling production of complex multicomponent vaccines poses logistical barriers related to process standardization, cost, and cold-chain requirements. Addressing these regulatory and manufacturing considerations is essential for ensuring that next-generation ESKAPE vaccines can advance successfully from laboratory innovation to widespread clinical deployment.

In conclusion, a sustained commitment to rational antigen selection, innovative vaccine technologies, and comprehensive evaluation is essential to develop cross-protective vaccines against ESKAPE pathogens. Such vaccines will be critical tools in reducing morbidity and mortality from multidrug-resistant infections and in curbing the global spread of antimicrobial resistance.

## Figures and Tables

**Table 1:** Summary of Vaccine Candidates Targeting ESKAPE Pathogens: Antigen Composition, Immunogenicity, Clinical Status, and Limitations.

Vaccine/Construct	Antigen Contents	Target Pathogen(s)	Protection/Immunogenicity	Clinical Stage	Limitations/Notes	
SA4Ag	ClfA, MntC, capsular polysaccharides CP5 & CP8	*S. aureus*	Immunogenic in preclinical studies; intended to elicit opsonic & neutralizing Abs	Phase 2b/3 (evaluated in surgical patients) Clinical trial identifier: NCT02388165	Failed to prevent surgery-associated *S. aureus* infections in trials; highlights translational gap.	[107] [34,108,109,110,111,112]
V710 (IsdB)	IsdB (iron surface determinant B)	*S. aureus*	Protected in some preclinical models (reduced bacterial burden)	Clinical trial (failed; associated with increased mortality in one trial) Clinical trial identifier: NCT00518687	Clinical safety signal and lack of efficacy underscore need for multi-antigen approaches.	[113,114]
LBT-SA7 (multivalent toxoid)	Multiple detoxified *S. aureus* toxins (multitoxoid formulation)	*S. aureus*	Toxoid strategy intended to neutralize secreted virulence factors; preclinical rationale strong	Phase 1 (ongoing ) Clinical trial identifier: NCT06719219	Promising novel approach; results pending.	
Sc(EH)_3_ (engineered AdcA epitopes)	Engineered epitopes from AdcA zinc-binding domain	*E. faecium* (cross-reactive with *S. aureus*)	Induces opsonophagocytic antibodies and conferred protection in murine systemic infection models	Preclinical	Demonstrates potential for cross-Gram-positive antigen design; needs broader validation.	[92]
*S. aureus* extracellular vesicle (EV) vaccine	Native EV protein cargo (multiple antigens)	*S. aureus*	Protective in murine pneumonia and lethal sepsis models; T cell–dependent protection reported	Preclinical	EVs provide multivalent native antigen display but require safety optimization and antigen mapping.	[50]
*K. pneumoniae* OMV vaccine	Native OMPs, LPS (modified), capsular components present in OMVs	*K. pneumoniae*	Recombinant OMVs demonstrated immunogenicity and protective efficacy in animal models	Preclinical	Capsular diversity and LPS reactogenicity are major design challenges.	
*P. aeruginosa* OMV vaccine	OprF, OprI in OMV preparations	*P. aeruginosa*	Protective in animal burn and infection models	Preclinical	OMV heterogeneity, endotoxin (LPS) toxicity, scale-up challenges; chronic infection & biofilm phenotypes complicate translation.	[39,60,61,62,63,115]
OprF/OprI hybrid protein vaccine (IC43)	Recombinant OprF–OprI fusion protein (Met-Ala-(His)_6_-OprF_190–342_–OprI_21–83_)	*P. aeruginosa*	Immunogenic in humans (induces OprF/OprI-specific IgG and opsonophagocytic activity). Also protective in animal models; improved protection when combined with PcrV in multivalent formats.	Early clinical/human Phase I safety & immunogenicity Clinical trial identifier: NCT00778388	Single-antigen limitations; broader or multivalent antigen combinations likely required for chronic or biofilm-associated disease.	[116,117,118]
PcrV-based vaccines/KB001 antibody	PcrV (T3SS tip protein); KB001 = anti-PcrV Fab	*P. aeruginosa*	Neutralizing antibodies/protection in animal models; KB001 detectable in human airways	Clinical testing in humans (no clinical benefit in CF patients reported) Clinical trial identifier: NCT01695343	Transient antigen expression, tissue penetration, and chronic infection biology limit efficacy.	[119]
Multi-epitope/in silico constructs	Variable—combinations of conserved epitopes (AdcA, Isd components, OMPs, siderophore receptors, OprF/I, etc.)	Multiple ESKAPE pathogens (species-dependent)	Predicted immunogenicity; several constructs elicited humoral & cellular responses in preclinical studies where tested	Mostly preclinical/computational designs; limited in vivo validation	Many constructs remain predictions—require experimental validation and standardization.	
LPS-deficient OMV vaccine (IB010)	Native OMVs from *A. baumannii* engineered to be LPS-deficient; contains multiple OMPs including OmpA and other vesicle-associated proteins	*Acinetobacter baumannii*	In BALB/c sepsis model, IM vaccination with 10 µg OMVs gave ~75% survival; 100 µg gave 100% survival. Vaccinated mice showed significantly reduced spleen CFU and decreased IL-1β/IL-6 levels	Preclinical (murine)	LPS reactogenicity addressed via LPS-deficient strain. Tested in sepsis—not respiratory—model. Strain breadth limited to study isolates.	[120]
Intranasal OMV vaccine	Whole OMVs (naturally containing OMPs including OmpA; not LPS-detoxified)	*Acinetobacter baumannii* clinical GC2 isolates	Intranasal immunization produced significant reduction in airway colonization and prevented systemic dissemination after intranasal challenge. IM vaccination produced antibodies but did not protect	Preclinical (murine)	Protection was route-dependent (only intranasal effective). Contains native LPS—reactogenicity unresolved. Data from limited GC2 isolates.	[59]
Recombinant BauA + OmpA subunit vaccine	Purified recombinant BauA (siderophore receptor) + OmpA	*Acinetobacter baumannii*	In murine sepsis model, combination immunization caused significant reductions in CFU in spleen, liver, and lungs versus controls and outperformed single-antigen groups. Authors report enhanced immunoprotection	Preclinical (murine)	Possible limited breadth because *A. baumannii* uses multiple iron-uptake pathways (siderophore redundancy). Tested with one primary clinical isolate	[98]
*K. pneumoniae*-derived extracellular vesicles (EVs)	Native EVs/OMVs containing outer membrane proteins (OMPs) (proteome analysis showed ~159 proteins) plus LPS and lipid-based vesicle structure.	*Klebsiella pneumoniae*	-In mice, three intraperitoneal immunizations with EVs (10 ng, 100 ng, or 1000 ng) induced high EV-specific IgG. -Splenocytes from immunized mice produced IFN-γ, IL-17, IL-4 after restimulation. -Survival: After challenge with lethal dose of *K. pneumoniae* (1 × 10^8^ CFU, intraperitoneal), 80% survival for 0.5 µg EV immunized mice, 100% survival for 1 µg EV immunized mice. -Adoptive transfer: both sera and splenocytes from EV-immunized mice conferred protection in naïve mice on challenge.	Preclinical (murine)	-Potential reactogenicity due to LPS in EVs.—The EVs were not engineered to remove or modify LPS.—Route used was intraperitoneal—translational relevance (e.g., human immunization route) uncertain.—Does not directly address capsule polysaccharide: EVs derived from *K. pneumoniae* may or may not contain capsule components in a way that elicits capsule-specific immunity.—Manufacturing and scale-up challenges (heterogeneity of EVs).	[55,121]
BSA-reinforced *K. pneumoniae* OMVs (BN-OMVs)	Hollow OMVs from *carbapenem-resistant K. pneumoniae*, internally reinforced by BSA (bovine serum albumin) nanoparticles, containing native OMPs and LPS.	*Klebsiella pneumoniae*, particularly carbapenem-resistant strains	-Subcutaneous vaccination in mice with BN-OMVs induced high CRKP (carbapenem-resistant *K. pneumoniae*)-specific antibody titers. -Protection: Mice immunized with BN-OMVs showed a significantly increased survival rate after lethal challenge with CRKP. -Adoptive transfer experiments showed the protective effect was dependent on both humoral and cellular immunity.	Preclinical (murine)	-The native LPS remains in OMVs—risk of endotoxin-mediated reactogenicity.—The nanoparticle (BSA) reinforcement improves stability but could raise regulatory or safety considerations.—Challenge model may use specific CRKP strain; may not fully represent diversity of *K. pneumoniae*.—Scale-up, reproducibility, batch heterogeneity of BN-OMVs may be problematic.	[122]

## Data Availability

Not applicable.

## Abbreviations

4C-Staph	Four-component *Staphylococcus aureus* vaccine
AIPs	Autoinducing peptides
AdcA	Adhesin zinc-binding lipoprotein A
AmpC	Class C β-lactamase
APC	Antigen-presenting cell
ATP	Adenosine triphosphate
Bap	Biofilm-associated protein
BPAg	Bacterial protective antigen
BSA	Bovine serum albumin
BepiPred	B-cell epitope prediction tool
cDNA	Complementary DNA
CD	Cluster of differentiation
CDS	Coding sequence
CF	Cystic fibrosis
ClfA	Clumping factor A
CMTR1	mRNA cap 2′-O-methyltransferase
CpG	Cytosine-phosphate-guanine motif
CP5/CP8	Capsular polysaccharide types 5 and 8
CW	Cell wall
DC	Dendritic cell
DNA	Deoxyribonucleic acid
DSPE-PEG	Polyethylene glycolated phospholipids
eDNA	Extracellular DNA
ELISA	Enzyme-linked immunosorbent assay
ESKAPE	*E. faecium*, *S. aureus*, *K. pneumoniae*, *A. baumannii*, *P. aeruginosa*, *Enterobacter* spp.
Esp	Enterococcal surface protein
ExoA	Exotoxin A
ExPEC10V	Extraintestinal pathogenic *E. coli* 10-valent vaccine
FnBP/FnBPA/FnBPB	Fibronectin-binding proteins
FOXP3	Forkhead box P3 protein
FpvA	Pyoverdine receptor A
FSAV / rFSAV	Five-antigen *S. aureus* vaccine
GMMA	Generalized modules for membrane antigens
GRAM	Global Research on Antimicrobial Resistance
HAI	Healthcare-associated infection
Hla	Alpha-hemolysin
Hcp1	Type VI secretion system component
HLA	Human leukocyte antigen
IC43	OprF-OprI hybrid *P. aeruginosa* vaccine
IFN-γ	Interferon-gamma
Ig/IgA/IgG/IgM	Immunoglobulins
IL	Interleukin
IM	Intramuscular
Isd/IsdA/IsdB	Iron-regulated surface determinants
KB001	Anti-PcrV monoclonal antibody
KLH	Keyhole limpet hemocyanin
*K. pneumoniae*	*Klebsiella pneumoniae*
LNP	Lipid nanoparticle
LPS	Lipopolysaccharide
LIA	Luminex immunoassay
MAP/MAP27	Multi-antigenic peptide
MDR	Multidrug resistant
MHC	Major histocompatibility complex
ML	Machine learning
mRNA	Messenger RNA
MrkD/MrkH	Fimbrial adhesin regulators
MSCRAMM	Microbial surface components recognizing adhesive matrix molecules
MVs	Membrane vesicles
NP	Nanoparticle
NCT	Clinical trial registry ID
NetMHCpan	MHC binding prediction tool
OMVs	Outer membrane vesicles
OMP	Outer membrane protein
OmpA/OmpK36/OmpW/Omp22/Omp34	Outer membrane antigens
OPK	Opsonophagocytic killing
PAMP	Pathogen-associated molecular pattern
PcrV	Type III secretion system tip protein
PGN	Peptidoglycan
PI3K	Phosphatidylinositol-3-kinase
PNAG	Poly-N-acetylglucosamine
Psl/Pel	*P. aeruginosa* exopolysaccharides
PLGA	Poly(lactic-co-glycolic acid)
QS	Quorum sensing
QSI	Quorum-sensing inhibitor
RNA-seq	RNA sequencing
RV	Reverse vaccinology
SA4Ag	*S. aureus* four-antigen vaccine
SagA	Secreted antigen A (*E. faecium*)
Sc(EH)_3_	Multi-epitope AdcA-derived antigen
SEB	Staphylococcal enterotoxin B
ST	Sequence type
Sta-V5	Five-antigen *S. aureus* vaccine
T3SS	Type III secretion system
Th/Th1/Th17	T helper cell subsets
TLR	Toll-like receptor
TNF-α	Tumor necrosis factor-alpha
TMB	Tetramethylbenzidine
UTR	Untranslated region
VRE/VREfm	Vancomycin-resistant *Enterococcus faecium*
VaxiJen	Antigenicity prediction tool
Vaxign2	ML-based vaccine design platform
V710	*S. aureus* IsdB vaccine
WHO	World Health Organization
WGS	Whole-genome sequencing
